# The segregated connectome of late-life depression: a combined cortical thickness and structural covariance analysis

**DOI:** 10.1016/j.neurobiolaging.2016.08.013

**Published:** 2016-12

**Authors:** Elijah Mak, Sean J. Colloby, Alan Thomas, John T. O'Brien

**Affiliations:** a Department of Psychiatry, University of Cambridge, Cambridge, UK; b Institute of Neuroscience, Newcastle University, Newcastle, UK

**Keywords:** Imaging, Depression, Dementia, Graph theory, Brain network, Late-life depression, Psychiatry

## Abstract

Late-life depression (LLD) has been associated with both generalized and focal neuroanatomical changes including gray matter atrophy and white matter abnormalities. However, previous literature has not been consistent and, in particular, its impact on the topology organization of brain networks remains to be established. In this multimodal study, we first examined cortical thickness, and applied graph theory to investigate structural covariance networks in LLD. Thirty-three subjects with LLD and 25 controls underwent T1-weighted, fluid-attenuated inversion recovery and clinical assessments. Freesurfer was used to perform vertex-wise comparisons of cortical thickness, whereas the Graph Analysis Toolbox (GAT) was implemented to construct and analyze the structural covariance networks. LLD showed a trend of lower thickness in the left insular region (*p* < 0.001 uncorrected). In addition, the structural network of LLD was characterized by greater segregation, particularly showing higher transitivity (i.e., measure of clustering) and modularity (i.e., tendency for a network to be organized into subnetworks). It was also less robust against random failure and targeted attacks. Despite relative cortical preservation, the topology of the LLD network showed significant changes particularly in segregation. These findings demonstrate the potential for graph theoretical approaches to complement conventional structural imaging analyses and provide novel insights into the heterogeneous etiology and pathogenesis of LLD.

## 1 Introduction

Late-life depression (LLD), often defined as depression in people over the age of 60, is common, and often associated with cognitive decline and future risk of dementia, increased disability, and mortality (Naismith et al., 2012). Estimates of the prevalence of clinically relevant depressive symptoms in older adults typically range from 10% to 15% and rates of major depression from 1% to 5%. Several diverse etiological factors have been proposed, including structural abnormalities due to vascular (Thomas et al., 2001) and neurodegenerative factors (Tsopelas et al., 2011), hypothalamo-pituitary-adrenal axis dysfunction and dysregulation of neurotransmitters such as serotonin (Meltzer et al., 1998).

Previous imaging studies have revealed a varied assortment of structural and functional abnormalities: localized gray matter atrophy in frontal cortex (Ballmaier et al., 2004) and subcortical structures (Colloby et al., 2011), increased distribution of white matter hyperintensities (WMHs) (Herrmann et al., 2008), microstructural deficits in white matter pathways (Sexton et al., 2012), and altered functional connectivity between subcortical regions (Kenny et al., 2010). However, the prevailing neuroimaging literature in LLD is still inconclusive. A meta-analysis of magnetic resonance imaging (MRI) studies in LLD only found weak evidence of hippocampal atrophy (7 of 15 studies) (Sexton et al., 2013), whereas others have not demonstrated any significant differences in gray matter (Colloby et al., 2011b, Koolschijn et al., 2010) or WMH (Colloby et al., 2011). A previous hypothesis-driven comparison of frontal lobar cortical thickness in this sample also did not show any significant differences compared with healthy controls (Colloby et al., 2011). These disparate findings could simply reflect the heterogeneity and the complex interaction of various factors in the pathophysiology of LLD, which might in turn obscure subtle disease-related alterations in the interaction patterns existing in large-scale networks of brain regions. In this regard, a multivariate technique might better explain the reported variability in neuroanatomical findings across studies compared with the conventional approach examining localized differences in discrete regions between groups.

In recent years, graph theoretical concepts have been increasingly applied to study the organizational principles of the brain by modeling it as a large-scale network with interconnected nodes and edges (Bullmore and Sporns, 2009). This framework rests on the fundamental premise that the maintenance or disintegration of complex systems is shaped by the interactions among their constituent elements. Bearing similarities to real-world scenarios such as the social network (i.e., 6 degrees of separation) and the cascading hyperlinks of the Internet, the human brain also possesses an inherent architecture known as the “small-world phenomenon” (Hagmann et al., 2008, Sporns et al., 2005). The small-world topology, with its short path lengths and high clustering (see Table 1 for a brief description of each network measure) supports efficient segregation and distribution of information processing with minimal cost (Bullmore and Sporns, 2009) and confers resilience against pathological damage (Achard et al., 2006). Conversely, deviations from small worldness toward randomization (shorter path lengths and lower clustering) or regularization (longer path lengths and higher clustering) have been found in the networks associated with neurodegenerative and psychiatric diseases, such as Alzheimer's disease (He et al., 2008), schizophrenia (Bassett et al., 2008), and major depressive disorder (Singh et al., 2013). The structural covariance method, referring to the coordinated variations in gray matter morphology (e.g., cortical thickness or volume), is increasingly used to infer structural connectivity between regions and construct large-scale brain networks (Alexander-Bloch et al., 2013). A key assumption underlying this methodology is that morphological correlations are related to some degree of axonal connectivity between brain regions with shared trophic, genetic, and neurodevelopmental influences (Alexander-Bloch et al., 2013). Although altered structural covariance networks have been found in a variety of brain diseases, it remains challenging to interpret disease-related changes in networks as we presently lack a clear understanding of the cellular and molecular mechanisms that drive the emergence of large-scale covariance across networks. Nevertheless, structural covariance networks derived from cortical thickness correlations have shown substantial agreement with white matter connections (Gong et al., 2012) and functional connectivity (Kelly et al., 2012).

To date, there have been very few studies assessing large-scale networks in LLD, yielding inconclusive evidence (See Table 2 for a literature summary). A recent diffusion tensor imaging (DTI) study of white matter connections identified longer path length and impaired global efficiency in LLD compared with controls (Bai et al., 2012). Using inter-regional correlations of gray matter volumes, another study in LLD also reported higher clustering in addition to longer path length (Ajilore et al., 2014), although no network differences were revealed by the same group in a subsequent analysis of white matter network on the same sample (Charlton et al., 2014). Preserved network organization in LLD has been reported in other studies using gray matter volumes (Lim et al., 2013) and functional data (Bohr et al., 2013). Furthermore, no study has performed a combined analysis of regional cortical thickness and network properties in the same sample, which will allow us to directly investigate the macro-level impact of cortical atrophy beyond the potentially affected regions.

The aims of this multimodal study are 3-fold: (1) we extended our previous frontal lobe study on this sample by employing a whole-brain vertex-wise approach to compare cortical thickness between LLD and controls; (2) from the regional thickness measures across the whole brain, we constructed a structural covariance network from the inter-regional correlations of cortical thickness to investigate global and regional properties of the LLD network; and (3) last, we investigated the resilience of both networks against random failures and targeted attacks. We hypothesized that LLD would be characterized by lower regional cortical thickness as well as aberrations in small worldness reflecting a shift toward a regularization of the network.

## 2 Method

### 2.1 Participants and clinical assessment

Subjects above the age of 60 years presenting to local psychiatry services with a history of a major depressive episode (Diagnostic and Statistical Manual of Mental Disorders [DSM-IV] criteria), current or previous were recruited. Specifically, the LLD group composed of participants who were still depressed (n = 16) as well as others who had remitted (n = 17). Healthy individuals were recruited via an advertisement placed in the local Elders Council magazine inviting participation to the study and all came from the same geographical area as the participants with depression. All participants and controls underwent the same set of assessments and structured interviews, although the controls did not do the mood rating scale. Thus, only healthy controls without a history of serious medical disorders such as stroke, diabetes, cancer, or other neurological diseases were recruited. Participants in the depression group were required to fulfill DSM-IV criteria for a lifetime diagnosis of major depressive episode.

A full neuropsychiatric assessment was conducted including family history of depression, previous psychiatric history, medical history, and current medication. Depression severity was rated using the Montgomery-Asberg Depressing Rating Scale (Montgomery and Asberg, 1979) and the 30-item Geriatric Depression Scale (GDS) (Yesavage et al., 1982). For all participants, the following exclusion criteria applied: dementia or mini-mental state examination score below 24, current use of a tricyclic antidepressant; comorbid or previous drug or alcohol misuse; previous head injury; previous history of epilepsy; previous transient ischemic attack or stroke; or a myocardial infarction within the previous 3 months.

### 2.2 Standard protocol approvals, registrations, and patient consents

The study was approved by the Newcastle and North Tyneside Research Ethics Committee. All subjects provided written informed consent.

### 2.3 MRI acquisitions: T1, fluid-attenuated inversion recovery, and DTI

Structural T1 imaging was performed using a 3T Achieva MR scanner (Philips Medical Systems, Eindhoven, The Netherlands). The T1-weighted volumetric sequence covered the whole brain (MPRAGE, sagittal acquisition, slice thickness = 1.2 mm, voxel size = 1.15 × 1.15 mm; repetition time = 9.6 ms; echo time = 4.6 ms; flip angle = 8°; sensitivity encoding [SENSE] factor = 2). The fluid-attenuated inversion recovery sequence was as follows: repetition time = 11000 ms, echo time = 125 ms, inversion time = 2800 ms, SENSE factor = 1.5, voxel size = 1.02 mm × 1.02 mm, 60 slices, slice thickness = 2.5 mm.

### 2.4 Image preprocessing

#### 2.4.1 Estimating cortical thickness from T1 MRI

Cortical reconstruction and volumetric segmentation of MRI data were performed using the Freesurfer image analysis suite (http://surfer.nmr.mgh.harvard.edu/) (Fischl and Dale, 2000, Fischl et al., 1999). The initial processing of T1 MRI images, for each subject and each time point (baseline and follow-up), includes the following steps: removal of nonbrain tissue, automated Talairach transformation, segmentation of the subcortical white matter and deep gray matter volumetric structures, intensity normalization, tessellation of the gray matter/white matter boundary, automated topology correction and surface deformation to optimally place the gray matter/white matter and gray matter/CSF boundaries. The cortical thickness was calculated as the closest distance from the gray/white matter boundary to the gray/CSF boundary at each vertex. The cortical thickness maps were smoothed using a 10-mm full width half maximum Gaussian. All surface models in our study were visually inspected for accuracy, and manual corrections were performed in the event of tissue misclassification/white matter errors while blinded to diagnostic group information. Ten subjects (5 controls and 5 LLD subjects) who had excessive pial or white matter surface segmentation errors after the manual correction were excluded from the analyses. Thus, the final sample was 25 controls and 33 LLD. To define the nodes for subsequent network analyses, the cortical thickness map of the cerebral cortex was parcellated using the Desikan-Killiany atlas, resulting in 34 regions of interest (ROI) for each hemisphere, each corresponding to the average cortical thicknesses of a gray matter region (Desikan et al., 2006).

#### 2.4.2 Quantifying WMHs

Volumetric measurements of global, periventricular, and deep WMH were obtained for each subject using a previously validated method. The technical details of this have been previously described (Colloby et al., 2011).

### 2.5 Vertex-wise comparisons of cortical thickness

Differences in regional cortical thickness between groups were assessed using a vertex-wise general linear model in Freesurfer QDEC. The model included cortical thickness as a dependent factor and diagnostic group (LLD and controls) as an independent factor. Age, gender, and cumulative illness rating scale for geriatrics (CIRS-G) were included as nuisance covariates. Correction for multiple comparisons was performed using false discovery rate (FDR) with significance threshold set at *p* < 0.05.

### 2.6 Structural covariance analyses

#### 2.6.1 Defining the nodes using inter-regional correlations of cortical thickness

**Fig. 1:**
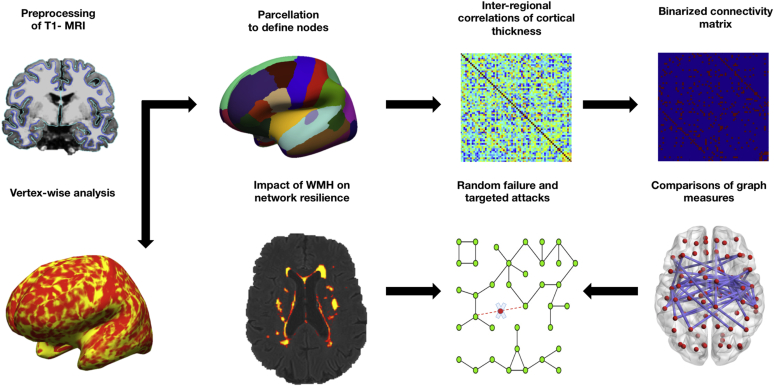
Analytical pipeline. Cortical reconstruction is processed on T1-weighted MRI with Freesurfer for 2 analytical streams: vertex-wise comparisons of cortical thickness maps between LLD and controls. For the network analyses, brain regions are assigned nodes according to definitions from the Desikan-Killiany parcellation scheme to yield the 68 × 68 association matrix. The inter-regional cortical thickness correlations are thresholded into a binary network containing only the strongest associations. Graph measures are calculated in GAT toolbox for statistical comparisons of network measures between LLD and controls. Resilience of the network was tested with random failures and targeted attacks (i.e., node removal based on betweenness centrality). The potential involvement of WMH on network resilience was investigated by including WMH volumes as an additional nuisance covariate in the resilience analyses. Abbreviations: GAT, Graph Analytical Toolbox; LLD, late-life depression; MRI, magnetic resonance imaging; WMH, white matter hyperintensities.

The full pipeline for the network analyses is illustrated in Fig. 1. To investigate the alterations in the architecture of structural networks in LLD compared with controls, we applied graph theoretical methods using the GAT (Hosseini et al., 2012), which integrates the Brain Connectivity Toolbox (Rubinov and Sporns, 2010) for the calculation and statistical comparisons of network measures. Specifically, networks were constructed for the LLD and control group using the structural covariance approach (Alexander-Bloch et al., 2013). The nodes in the network correspond to the 68 cortical ROIs extracted from the Desikan-Killiany atlas. Consistent with previous studies, linear regression was performed at each ROI to remove the effects of covariates, including age, gender, CIRS-G, and mean cortical thickness (Bernhardt et al., 2011, He et al., 2007). The resulting residuals of this regression are then substituted for the unadjusted cortical thickness at each ROI. Therefore, the structural covariance networks for the LLD and controls group were constructed based on a 68 × 68 association matrix, with each entry defined as the Pearson’s correlation coefficient *R* between every pair of ROI.

#### 2.6.2 Defining the edges through thresholding

From the association matrix for each group, a binary matrix is derived after thresholding, where an entry is 1 if *R* is greater than a minimum density threshold in each group. Consistent with previous studies (Hosseini et al., 2012), we thresholded the association matrices at a range of network densities, from a minimum density of 10%–20% in steps of 1%. This was done to ensure that group differences are not confounded by differing number of nodes and edges due to an absolute threshold at a single density. The density of a network relates to the fraction of edges present in the network compared with the maximum possible number of edges. The minimum density (D_*min*_) is the density at which all the nodes are fully connected in the network of each group. This ensures that none of the networks are fragmented. The diagonal elements of the association matrix (i.e., self-connections) are set to 0. The resultant adjacency matrix represents a binary undirected graph. After generating the structural covariance networks of LLD and controls, we compared the network measures of interest across the range of densities. These measures include small worldness, characteristic path length, global efficiency, clustering coefficient, local efficiency, transitivity, and modularity. Brief descriptions of the network measures in this study are provided (Table 1).

#### 2.6.3 Statistical comparisons of network measures between LLD and controls

The binarized adjacency matrices are then estimated by applying the same thresholding procedure as previously described. To test the statistical significance of the between-group differences in network measures, nonparametric permutation tests with 1000 repetitions were performed in GAT. In conjunction with permutation testing, area under a curve (AUC) analyses was implemented to compare the curves depicting changes in a specific network measure (for each group) as a function of network density (Hosseini et al., 2012). Each of these curves depicts the changes in a specific network measure as a function of network density. The significance of the between-group differences in the AUC of each measure was similarly tested with a permutation analysis as described (Hosseini et al., 2012). A key advantage of this secondary approach is that by providing a summary *p*-value of difference, the comparison between network measures is less sensitive to the thresholding process.

#### 2.6.4 Investigating network resilience to random failure and targeted attacks

To assess the resilience of brain networks in LLD and controls to acute and focal damage, networks can be lesioned by random deletions of nodes or by targeted attack based on the highest degree or clustering of a node (Achard et al., 2006, Joyce et al., 2013, Váša et al., 2015). Random failure of the networks was simulated by randomly removing 1 node from the network. The impacts of these computational insults in both LLD and control networks were quantified by measuring the relative changes in the size of the largest remaining component. The largest remaining component in a network refers to a subgraph in which any 2 vertices are connected via edges, and which is not connected to the rest of the graph. To assess the network behavior against targeted attacks, the same procedure was applied by removing nodes in rank order of decreasing betweenness centrality, a measure of the number of the shortest paths that pass through 1 node. The removal of nodes on the basis of betweenness centrality is a suitable paradigm for the assessment of network robustness because it characterizes the relative influence of a brain region/node for integration of information across multiple brain regions. Finally, to test the differential responses of the networks in each group against random failure and targeted attacks, a permutation analysis was performed as previously described. The comparisons of network resilience were made at D_*min*_, the lowest density at which all regions were fully connected in both networks. This ensured the involvement of all regions in the network model without extraneous connections that could confound the results of subsequent network failure analyses.

#### 2.6.5 Qualitative hub analyses

We also performed a descriptive analysis of the spatial distribution of hubs in the networks of controls and LLD. Hubs are crucial components for efficient communication in a network as they are usually traversed by a large number of shortest paths between pairs of nodes (Bullmore and Sporns, 2009). In healthy controls, hubs have been found within highly connected association cortex, whereas previous studies have also found altered distribution of hubs in neurodegenerative conditions such as AD (He et al., 2008). A region or node is considered a hub if its betweenness centrality is 2SD greater compared with the network.

### 2.7 Statistical analyses

Statistical analyses were performed with the STATA13 (http://www.stata.com/) software. Distribution of continuous variables was tested for normality using the Skewness-Kurtosis test and visual inspection of histograms. Parametric data were assessed using either *t*-tests or analysis of variance (ANOVA) for continuous variables. For nonparametric data, Wilcoxon rank-sum test or Kruskal–Wallis test was used. χ^2^ tests were used to examine differences between categorical variables. Analysis of covariance was used to compare the distribution of WMH, accounting for age, gender, and intracranial volumes. For each test statistic, a 2-tailed probability value of <0.05 was regarded as significant.

## 3 Results

### 3.1 Sample characteristics and clinical features

Demographics, clinical characteristics, and imaging measures of the sample are shown in Table 3. Both the LLD group and controls were well matched for age (*p* = 0.960) and gender (*p* = 0.746). As might be expected, the LLD group scored significantly lower on mini-mental state examination (*p* = 0.006), although both were within the normal range. Seventeen LLD subjects were in remission. There was no significant age (*p* = 0.326) or gender (*p* = 0.362) difference between those in remission compared with those who were not in remission. As expected, GDS scores were lower in the remission group (*p* < 0.001). Seventeen subjects had early-onset depression. No differences in age (*p* = 0.135), gender (*p* = 0.362), and GDS (*p* = 0.793) were found between early-onset and late-onset groups. The LLD group also had significantly higher CIRS-G scores, but this was due mainly to differences in genitourinary symptoms (*p* < 0.001).

### 3.2 Cortical thickness comparisons

**Fig. 2:**
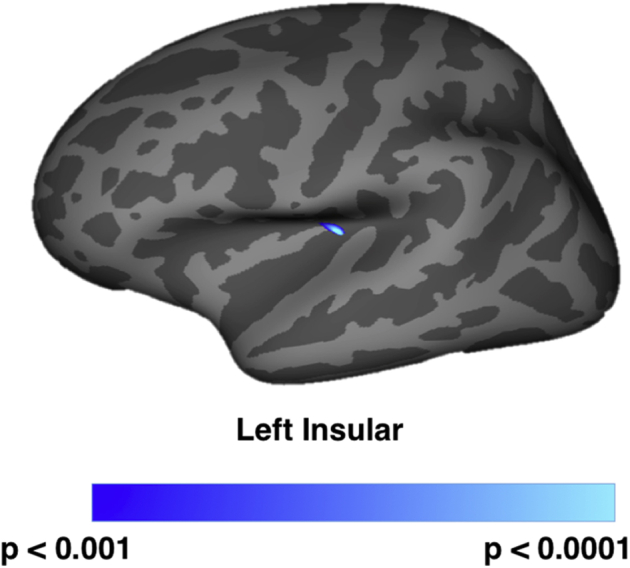
Vertex-wise comparisons of cortical thickness between LLD and controls. After correcting for age, gender, and CIRS-G, cortical thickness was lower and was observed in the left insular of the LLD compared with controls at *p* < 0.001 (uncorrected for multiple comparisons). Abbreviations: CIRS-G, cumulative illness rating scale for geriatrics; LLD, late-life depression.

Global cortical thickness did not significantly differ between LLD and controls (*p* = 0.341) (Table 3). Similarly, the vertex-wise comparisons with correction for multiple comparisons of cortical thickness found no differences between both groups. However, at a liberal threshold of *p* < 0.001 (uncorrected), the LLD group showed a focal reduction of cortical thickness in the left insular compared with healthy controls (Fig. 2).

### 3.3 White matter hyperintensities comparisons

No differences were found between LLD and controls in all measures of WMH, including total WMH (*p* = 0.730), periventricular WMH (*p* = 0.991), and deep WMH (*p* = 0.534) (Table 3).

### 3.4 Structural covariance network analyses

**Fig. 3:**
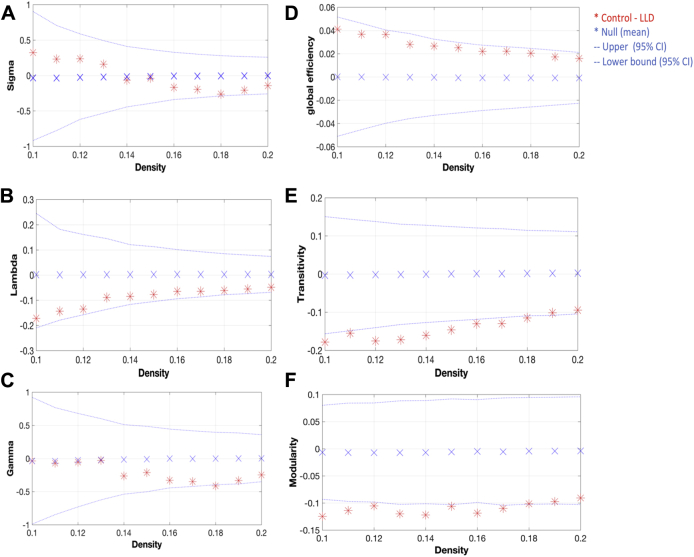
Between-group differences in global network topology as a function of network density. (A) Small-world index, (B) Lambda (normalized characteristic path length), and (C) Gamma (normalized clustering coefficient); (D) clustering coefficient, (E) transitivity coefficient, and (F) modularity. The red * marker represents the difference between LLD and controls network (+ve = controls > LLD; −ve = LLD > controls), with those positioned out of the confidence intervals representing significant differences after permutation testing with 1000 repetitions (*p* < 0.05). Abbreviation: LLD, late-life depression. (For interpretation of the references to color in this figure legend, the reader is referred to the Web version of this article.)

The minimum density below which the networks in both groups were fragmented was D_*min*_ = 0.1. The networks of both groups showed small-world organization across a wide range of densities (small world index >1); suggesting that both networks had a path length slightly higher than random networks, whereas having a clustering coefficient much higher than that of a random network. We investigated between-group differences in global network measures on networks thresholded across a range of densities (0.1:0.01:0.2) (Fig. 3). Although the LLD group showed longer lambda values (normalized characteristic path length) and lower global efficiency, this difference was not significant across the range of network densities. However, the LLD network had significantly greater transitivity and modularity across the range of network densities. A subsequent AUC analysis consistently showed higher transitivity (*p* = 0.025) and modularity (*p* = 0.022).

#### 3.4.1 Regional network characteristics

We investigated both the networks (density = 0.1) for between-group differences in regional network measures, such as nodal betweenness, nodal clustering, and nodal degree. No significant differences in nodal characteristics were found after correction for multiple comparisons across the 68 ROIs.

#### 3.4.2 Network resilience against random failure and targeted attacks

**Fig. 4:**
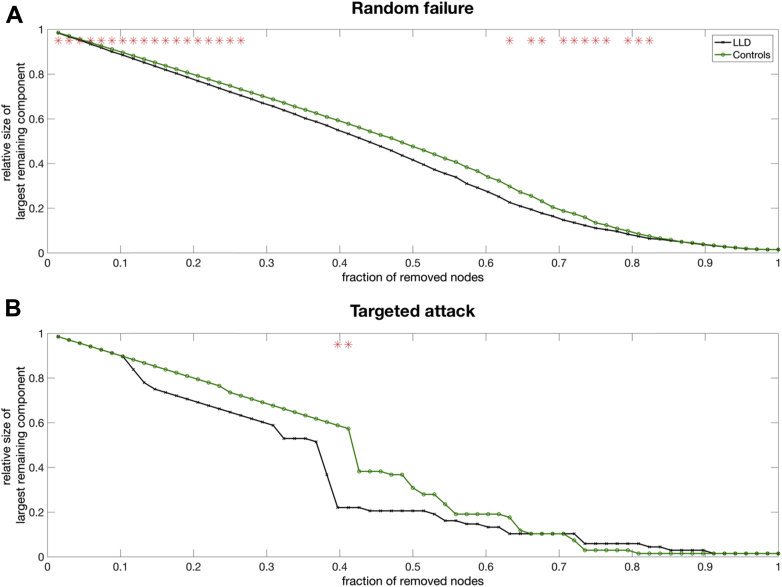
Comparison of network resilience. Changes in the size of the largest component of the remaining network after (A) cascading random failure and (B) targeted attack in order of nodal betweenness. The red * marker represents significant differences in the size of the largest remaining component between LLD and controls. Abbreviation: LLD, late-life depression. (For interpretation of the references to color in this figure legend, the reader is referred to the Web version of this article.)

Compared with the controls, the LLD network showed less tolerance to random failures (i.e., smaller size of the largest remaining component) at most fractions of removed node (Fig. 4). To a smaller extent, the LLD network was also more vulnerable to targeted attacks by removal of nodes in rank order of decreasing betweenness centrality, showing significant fragmentation at 0.4 fraction of node removal. The LLD network was still more vulnerable to random failure after accounting for WMH as an additional covariate, whereas the between-group difference in robustness to targeted attacks was attenuated, and no longer significant.

#### 3.4.3 Identification of hubs

**Fig. 5:**
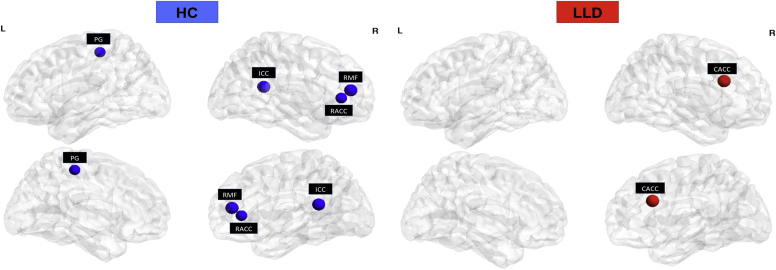
Spatial distribution of hubs in the structural covariance networks of controls and LLD. Abbreviations: CACC, caudal anterior cingulate cortex; ICC, isthmus cingulate cortex; LLD, late-life depression; PG, paracentral gyrus; RACC, rostral anterior cingulate cortex; RMF, right rostral middle frontal cortex.

The illustration of hubs in both groups is shown in Fig. 5. In the networks thresholded at D_*min*_, we considered a node in a hub if its betweenness centrality is 2SD higher than the mean betweenness centrality in the network (He et al., 2008). The hubs in the control network were found in the left paracentral gyrus, right isthmus cingulate cortex, right rostral anterior cingulate cortex, and right rostral middle frontal cortex. In the LLD network, only the right caudal anterior cingulate cortex was identified as a hub.

## 4 Discussion

This was the first study to perform a combined analysis of regional cortical thickness and large-scale network properties in LLD. Our findings were partially consistent with our primary hypotheses. Despite showing a relative cortical preservation, the LLD network was characterized by higher segregation as reflected by greater transitivity and modularity compared with controls. This deviation from an optimal small-world architecture was also accompanied by lower resilience to random failure and targeted attacks, the latter of which could be partially mediated by WMH. These results collectively suggest an altered topology of structural covariance networks in patients with LLD.

Previous ROI and voxel-based morphometry analyses on this sample by our group have demonstrated preserved frontal lobar cortical thickness and gray matter volumes, respectively, (Colloby et al., 2011). The present study extended the analyses to perform a whole-brain, vertex-wise comparison of regional cortical thickness, and no significant reductions of cortical thickness were found in LLD. Although this observation sits in contrast to a meta-analysis revealing widespread gray matter reductions in LLD (Sexton et al., 2013), our negative finding is consistent with a previous study comparing 28 female subjects with LLD with 38 age-matched controls (Koolschijn et al., 2010). The relative absence of focal gray matter abnormalities has prompted us to argue that white matter pathology could be more pertinent to the neurobiology of LLD. Indeed, a previous DTI–tract based spatial statistics (TBSS) analysis on the same sample suggested subtle deficits of integrity in white matter fibers within frontal, temporal, and midbrain regions (Colloby et al., 2011). In addition to microstructural alterations in white matter, the pathophysiological background of LLD is complicated by a host of factors, including vascular, neurotransmitter disruptions, and amyloidosis reminiscent of Alzheimer's disease pathology (Nascimento et al., 2015). Collectively, the broad range of changes in LLD has given rise to the notion that LLD could be characterized as a systems-level disorder, one that is particularly suited to be investigated by a multivariate network approach that considers the orchestrated interactions between distinct neuroanatomical regions.

In this study, the structural networks in LLD and controls were characterized by a small-world architecture that was not significantly different between groups. However, the small worldness of a brain network is determined by its underlying attributes such as segregation and integration. Although the LLD network showed longer characteristic path length and lower global efficiency, this difference did not reach statistical significance. A similar nonsignificant trend for longer path lengths has been previously reported in another sample of LLD (Ajilore et al., 2014), in addition to other significant findings of network disintegration that have been reported in other studies of LLD (Bai et al., 2012, Li et al., 2015) and AD (Dai and He, 2014, He et al., 2008). On the other hand, segregation reflects the capacity to perform specialized processing within a clique of densely interconnected brain regions. The LLD network in the present study had greater transitivity and a higher degree of modularity, both of which have not been previously reported in the LLD graph theory literature (Table 2). The concept of modularity is increasingly popular in graph theory research, which posits that brain networks can be decomposed into classes of modules or subnetworks. As such, it is also another index for the degree to which the brain is compartmentalized. Indicative of an abnormally strong local specialization and segregation, our paired findings of higher transitivity and modularity could suggest that information processing in the LLD brain network is traversing restrictedly within a clique of densely interconnected regions.

However, in contrast to the well-established findings of lower integration in LLD, there is less agreement about the presence and direction of changes in the segregative properties of LLD networks: both higher (Ajilore et al., 2014) and lower (Li et al., 2015) clustering have been reported from structural and functional networks respectively. In parallel, a similar dichotomy of between-group differences in clustering has emerged in the AD graph theory literature, where structural and functional networks have demonstrated higher (Daianu et al., 2015, He et al., 2008, Yao et al., 2010) and lower (de Haan et al., 2009, Stam et al., 2009, Supekar et al., 2008) clustering respectively. The reconciliation of seemingly disparate findings from structural and functional networks represents a critical challenge in the rapidly growing field of graph theory, although it could also serve to suggest that each imaging modality could be characterizing unique information about the human connectome albeit from different perspectives.

It is tempting to hypothesize that the segregated topology of the LLD network might render it less resilient to network dysfunction due to its failure to recruit alternative routes for information pathways (i.e., parallel processing) (Achard et al., 2006). In agreement with previous studies in LLD (Ajilore et al., 2014) and AD (He et al., 2008), both our findings support this hypothesis. The LLD networks showed a significantly greater degree of fragmentation when subjected to both random removal and targeted removals of nodes based on betweenness centrality. Considering the heterogeneous etiology of LLD, the potential involvement of vascular pathology to the weakened robustness of the LLD network was also investigated by accounting for WMH in a secondary analysis. Although the LLD network was still showing less resilience to random attacks, between-group differences in robustness to targeted attacks were attenuated and no longer significant. This suggests that, instead of a diffuse effect of topological destabilization, WMH could be locally deleterious to highly influential nodes that serve as bridges between disparate components of a brain network. It would be desirable to test this hypothesis using single-subject connectivity matrices from resting-state functional MRI or diffusion tractography datasets.

There was a distinct distribution of hubs in both LLD and control networks. Specifically, a loss of hubs in frontal and posterior cortices was found in LLD compared to controls. LLD network showed which could, in turn, compromise the topological stability of the networks to both random and targeted attacks as described. Given their centrality to the networks, hubs are biologically costly and vulnerable to disease-related processes (Crossley et al., 2014). In this context, local blood flow of a node could represent a surrogate measure of “biological cost”. Indeed, hypoperfusion in frontal regions is a consistent finding in LLD (Awata et al., 1998). Future studies are needed to further investigate the effects of hypoperfusion on network measures at the individual level.

With the repertoire of analyses across network measures, WMH-related network vulnerability, and hub analyses, we could attempt to offer some tentative insights into the potential factors leading to the segregated adaptation of the LLD network observed in this study. First, we have shown that WMH could account for network vulnerability against targeted attacks of nodes with high betweenness centrality, otherwise known as hubs. WMH and other ischemic changes are preferentially distributed within the periventricular and deep white matter regions, leading to disruptions of long projection fibers that are crucial for signal propagation across longer distances. The deletion of these “network shortcuts” may induce a rerouting of network communications by forcing signal propagation to traverse neighboring and adjacent circuits, in turn increasing the segregation within the network. Second, the loss of frontal and posterior hubs in LLD could also lead to a fragmentation of the LLD network into dense clusters that are highly intraconnected but weakly interconnected with other clusters of regions.

There are several strengths to our multimodal study. To the best of our knowledge, this was the first study to compare regional cortical thickness and network measures in the same sample. Unlike the Voxel-based morphometry (VBM) approach in previous studies (Lim et al., 2013), we used regional cortical thickness to construct our structural covariance networks, thereby overcoming the main limitation of VBM, providing a mixed measure of the cortical gray matter, including surface area, cortical folding, as well as cortical thickness (Hutton et al., 2009). The structural covariance approach also offers another alternative approach that could sidestep the limited validity of DTI-based approaches to map cortico-cortical connectivity due to the multitude of branching and crisscrossing fibers. However, the structural covariance networks constructed in the present study is estimated on the basis of inter-regional correlations at a group-level (LLD and controls) and does not provide individual networks for each subject, precluding correlational investigations with clinical measures, such as WMH burden as described previously. Our study also benefited from participants being clinically assessed by a psychiatrist. We used robust and validated methods for our imaging techniques, and the sample was reasonably sized compared with similar studies in late-life depression. A potential limitation was that we did not control for the potential effects of medication on the structural networks. It is possible that psychotropic medications could influence results but other medications typical in older populations such as anti-hypertensives and statins are less likely. However, because medications were too diverse in the depressed cohort, a rigorous statistical evaluation was impractical. Our ability to identify cortical changes might have been limited by the wide range of participants recruited, such as the grouping of LLD subjects both in remission and nonremission. However, we believe our findings are generalizable in light of the heterogeneous nature of LLD (Ajilore et al., 2014). Furthermore, finally, although the number of graph metrics examined in this study was comparable to the literature, there could still be a risk of type 1 error. Future studies with larger sample size are warranted to confirm our novel findings of higher transitivity and modularity in LLD networks. With regards to the image processing, 10 subjects were excluded due to segmentation errors that could not be adequately corrected. This is similar to other studies with a failure rate of 10%–15% (Watson et al., 2015). The performance of Freesurfer could be improved by averaging across multiple T1 scans per subject due to improvements in motion correction. This could be a consideration for future studies particularly in data sets involving elderly patients.

In this study, graph theoretical analyses revealed global network disruptions in LLD despite comparable cortical thickness to that of healthy controls. The LLD network was highly segregated with significantly higher transitivity and modularity, both of which have been consistently reported in neurodegenerative conditions such as AD. These topological disruptions were accompanied by more vulnerability to network disturbances, which in turn, could be accounted by the presence of WMH. Taken together, these network disturbances provided early evidence that graph theory is a promising framework to investigate the heterogeneous etiology and pathogenesis of LLD, although further studies in this growing field are warranted.

## Disclosure statement

Alan Thomas reports grants from the Medical Research Council, NIHR, ARUK, and the Alzheimer's Society and has received funding from GE Healthcare for investigator led research. John O'Brien reports grants from the Medical Research Council, NIHR, ARUK, and the Alzheimer's Society and has acted as a consultant for GE Healthcare, Lilly, TauRx, Axona, and Cytox. Elijah Mak and Sean Colloby have no competing interests.

## Figures and Tables

**Table 1:** Description of network measures investigated in this study

Small worldness is a measure of how much a network is locally interconnected compared against a random network while retaining efficient global connectivity between distant brain regions. Thus, its main attributes are a higher clustering coefficient but a similar characteristic path length compared with that of a random network.			
Integration		Segregation	
Characteristic path length	The shortest path length is the smallest number of connections to get from one node to another. The characteristic path length is the average of the shortest path length between all the pairs of nodes in the network. It is the most commonly used measure of network integration.	Clustering coefficient	The clustering coefficient of a node is a measure of the number of edges that exist between its nearest neighbors. The clustering coefficient of a network is thus the average of clustering coefficients across the nodes.
Global efficiency	The global efficiency is the average of the inverse shortest path length in the network.	Transitivity	Often used as an alternative to clustering coefficient, transitivity reflects the likelihood for a network to have interconnected nodes that are adjacent to one another, and is normalized by the whole network. It is also more robust compared to clustering coefficient, as it is not influenced by nodes with small number of connections (Newman, 2003).
		Modularity	The extent to which a network is characterized by densely interconnected nodes with relatively few connections between nodes in different modules (“cliques”). It is a reflection of the natural segregation within a network.
		Local efficiency	The local efficiency refers to the global efficiency of the subgraph (i.e., fully connected network not connected to the main graph) formed by the adjacent neighbors of the node.

**Table 2:** Literature summary of principal findings in network studies in LLD across imaging modalities

Study	Modality	Groups	Findings: LLD versus Controls
Ajilore et al., 2014	3T MRI (gray matter volumes)	53 LLD and 73 controls	Higher clustering coefficient and path lengths at trend levels. Lower global efficiency. No difference in resilience to random failure, more vulnerability to targeted attacks based on nodal influence.
Lim et al., 2013	3T MRI (gray matter volumes)	37 LLD and 40 controls	No differences in clustering coefficient, path length, and small-world index. Lower nodal betweenness in the medial orbitofrontal and angular gyrus regions.
Bai et al., 2012	DTI	35 RGD, 38 aMCI, and 30 controls	Both RGD and aMCI showed longer path length and lower global efficiency. No differences between RGD and aMCI.
Charlton et al., 2014	DTI	28 LLD and 48 controls	No differences in global measures. Higher vulnerability in the right prefrontal cortex; lower centrality in the right temporal region.
Bohr et al., 2013	Resting-state fMRI	14 LLD and 16 controls	No differences in global measures.
Li et al., 2015	Resting-state fMRI	23 LLD, 18 aMCI, 13 LLD + aMCI, and 25 controls	LLD+aMCI showed longer path length and lower global efficiency. Both LLD groups showed lower local efficiency.

Key: aMCI, amnestic mild cognitive impairment; DTI, diffusion tensor imaging; LLD, late-life depression; MRI, magnetic resonance imaging; RGD, remitted geriatric depression.

**Table 3:** Values expressed as mean ± standard deviation

	Controls	LLD	*p*-value
n	25	33	
Age (y)	73.6 ± 6.0	73.6 ± 5.2	0.96^t^
Age range	61–80	60–84	
Gender (male, %)	7 (28%)	8 (24.24%)	0.75^§^
Disease duration (mo)		22.6 ± 20.7	NA
Onset (y)		51.0 ± 22.1	NA
MMSE	29.6 ± 0.8	28.9 ± 1.0	0.01^w^
MADRS		13.5 ± 10.9	NA
GDS		12.4 ± 8.1	NA
CIRS-G	3.6 ± 1.5	6.4 ± 2.6	<0.001^t^
Mean cortical thickness (mm)	2.29 ± 0.08	2.26 ± 0.13	0.34^a^
Total WMH (mL)	10.3 ± 15.5	8.4 ± 8.3	0.73^a^
Pv WMH (mL)	6.7 ± 7.8	6.1 ± 5.5	0.99^a^
Deep WMH (mL)	3.6 ± 8.7	2.2 ± 3.4	0.53^a^

Significance set at *p* < 0.05. The superscripts, “t” represents Student's *t*-test; “w” represents Wilcoxon Ranksum; “§” represents χ^2^ test; “a” represents analysis of variance.

Key: CIRS-G, cumulative illness rating scale for geriatrics; GDS, Geriatric depression scale; LLD, late-life depression; MADRS, Montogomery-Asberg depression rating scale; MMSE, mini-mental state examination; NA, not available; Pv WMH, periventricular WMH; WMH, white matter hyperintensity.
